# Retraction: Down-regulation of microRNA-320 suppresses cardiomyocyte apoptosis and protects against myocardial ischemia and reperfusion injury by targeting IGF-1

**DOI:** 10.18632/oncotarget.28704

**Published:** 2025-03-13

**Authors:** Chun-Li Song, Bin Liu, Hong-Ying Diao, Yong-Feng Shi, Ji-Chang Zhang, Yang-Xue Li, Ning Liu, Yun-Peng Yu, Guan Wang, Jin-Peng Wang, Qian Li

**Affiliations:** ^1^Department of Cardiology, The Second Hospital of Jilin University, Changchun 130041, P. R. China

**This article has been retracted:** Oncotarget has completed its investigation of this article. Several instances of internal and external image overlaps and duplications were discovered. Specifically, Figure 2, illustrating the efficiency of different viruses transduction into myocardial tissues, contains overlaps in panels A, B and C. Figure 7A, showing data of flow cytometry, has two duplicated images which should represent different experimental conditions. Figure 7A also has images found in Figure 4 of an unrelated earlier published paper, which has been retracted [1], and Figure 3A of [2]. Western blot images of b-actin in Figures 6C, 8A and 9A were found in an earlier published paper [3] and in papers published concurrently [4]. In addition, the already retracted paper [5] shared the western blot images with Figure 7C and 9A. While the corresponding author Chun-Li Song provided a corrected Figure 7, the other concerns remained unaddressed. Additionally, the author indicated unresolved authorship disputes and requested the manuscript’s withdrawal. This retraction request was also acknowledged by The Second Hospital of Jilin University. Given these findings and the authorship dispute, the editorial decision has been made to retract the paper. All authors have agreed with this decision.

Original article: Oncotarget. 2016; 7:39740–39757. 39740-39757. https://doi.org/10.18632/oncotarget.9240
